# Preemptive Duloxetine Relieves Postoperative Pain and Lowers Wound Temperature in Centrally Sensitized Patients Undergoing Total Knee Arthroplasty: A Randomized, Double-Blind, Placebo-Controlled Trial

**DOI:** 10.3390/jcm10132809

**Published:** 2021-06-25

**Authors:** Man Soo Kim, In Jun Koh, Yong Gyu Sung, Dong Chul Park, Jae Won Na, Yong In

**Affiliations:** 1 Department of Orthopedic Surgery, Seoul St. Mary’s Hospital, College of Medicine, The Catholic University of Korea, 222, Banpo-daero, Seocho-gu, Seoul 06591, Korea; kms3779@naver.com (M.S.K.); ygsung@catholic.ac.kr (Y.G.S.); dc1225@naver.com (D.C.P.); lapiki427@gmail.com (J.W.N.); 2 Department of Orthopedic Surgery, Eunpyeong St. Mary’s Hospital, College of Medicine, The Catholic University of Korea, 1021, Tongil Ro, Eunpyeong-gu, Seoul 03312, Korea; oskoh74@gmail.com

**Keywords:** duloxetine, pain, wound healing, central sensitization, total knee arthroplasty, randomized

## Abstract

(1) Background: The purpose of this study was to determine whether preemptive duloxetine in patients with central sensitization (CS) is effective for acute postoperative pain control and wound healing following total knee arthroplasty (TKA). (2) Methods: CS was defined as a score of 40 points or higher on the Central Sensitization Inventory (CSI) survey. Thirty-nine patients with CS were randomly assigned to either the duloxetine group (*n* = 19) or the placebo group (*n* = 20). The duloxetine group took duloxetine 30 mg once a day, while the placebo group took the placebo medication once a day. A pain visual analog scale (VAS) and the Brief Pain Inventory (BPI), wound complications, the temperature of the surgical site, and adverse events were investigated. Skin temperature was measured at the center of the patella using a portable digital thermometer. (3) Results: The duloxetine group reported significantly lower pain VAS scores during follow-up periods up to 6 weeks after surgery (all *p* < 0.05). BPI interference also showed significantly superior results in the duloxetine group after surgery (all *p* < 0.05). Although there was no difference in the rate of wound complications between the two groups (*p* > 0.05), the duloxetine group showed significantly lower wound temperature than the placebo group during the follow-up period (all *p* < 0.05). (4) Conclusion: In this study, preemptive duloxetine effectively reduced pain and lowered wound temperature following TKA in CS patients.

## 1. Introduction

Total knee arthroplasty (TKA) is the most effective treatment for end-stage knee osteoarthritis (OA) [1]. It is an established surgical method that reduces pain and provides a better quality of life through functional enhancement. However, about 20% of patients who undergo TKA are not satisfied with the results of the operation due to persistent pain [2,3]. Perioperative pain is closely related to surgical stress response [4]. Various pain control methods have been used to reduce surgical stress response as well as perioperative pain [5,6]. Moreover, despite the remarkable developments in multimodal pain analgesia in recent years, pain control after TKA surgery remains challenging.

Central sensitization (CS) has received significant attention recently as the cause of such persistent pain [7,8]. CS appears due to an abnormal reaction of the central nervous system and is characterized by allodynia and hyperalgesia [7,8]. CS is present in 20% to 40% of patients with chronic knee pain with advanced knee OA [9,10,11]. Many studies have assessed the effect of CS on the clinical manifestations that appear after TKA. Preoperative CS is well known as a risk factor for persistent pain and inferior function after TKA [12,13,14,15]. In CS patients, the function of the serotonin–norepinephrine descending inhibitory pathway decreases, resulting in a decrease in serotonin and norepinephrine [16,17]. Serotonin and norepinephrine are closely related to the inflammatory and proliferative phases of wound healing and act as important neurotransmitters in wound healing [18,19,20]. CS is thus closely related to wound complications after TKA [21]. Proper wound healing after TKA is important for patient recovery, rehabilitation, and the prevention of periprosthetic joint infection [22,23]. Wound complications after TKA are an important risk factor for the exacerbation of deep periprosthetic joint infections [22,23].

Duloxetine (Cymbalta; Eli Lilly & Co., Indianapolis, IN, USA) is a selective serotonin–norepinephrine reuptake inhibitor (SNRI) that acts on the descending inhibitory pain pathway in the central nervous system [24,25]. The analgesic effect of duloxetine is well established in patients with central mediated musculoskeletal pain, including those with pain from chronic knee OA [26]. In the context of TKA, duloxetine has also been found to be excellent for reducing pain and facilitates a superior quality of recovery among CS patients [13]. In addition, duloxetine might play an important role in reducing wound complication rates in CS patients undergoing TKA [21]. However, the pain reduction effects of duloxetine after TKA only appear 2 weeks after surgery [13]. SNRIs exhibit potent antiplatelet and endothelium protective effects, exacerbate the development of inflammation, and control the production of interleukin and interferon to help wound healing [27].

No prospective study has examined whether duloxetine can reduce postoperative pain and the incidence of wound complications in CS patients undergoing TKA [13,21]. The purpose of this interim study was to investigate whether preemptive duloxetine is effective in reducing immediate postoperative pain following TKA and in supporting improved wound healing in CS patients. We hypothesized that preemptive duloxetine is effective in reducing pain and aiding in wound healing in CS patients undergoing TKA.

## 2. Methods

**Figure 1:**
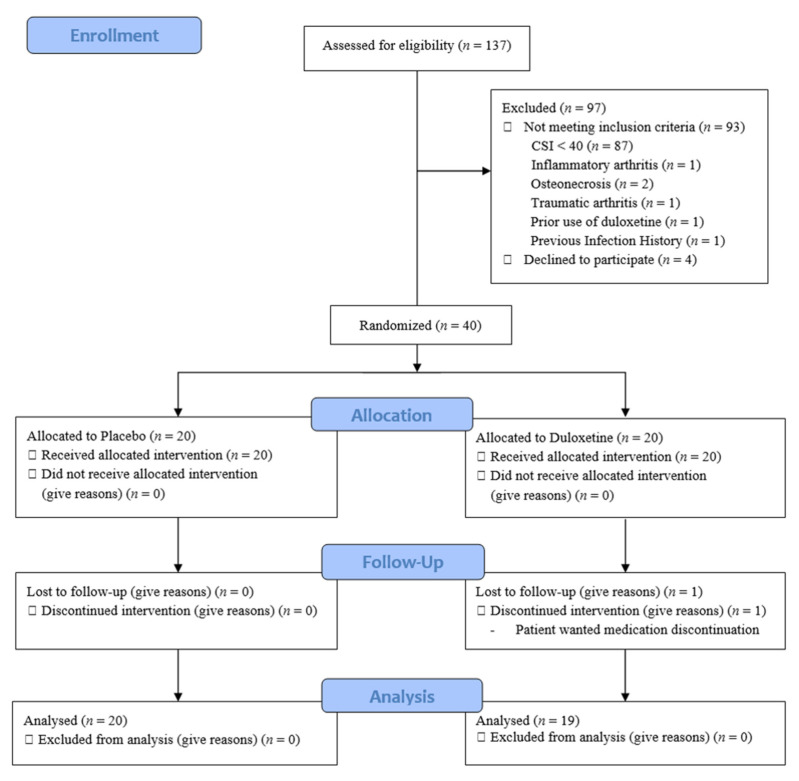
Flow of patient enrollment, randomization, and follow-up. CSI, Central Sensitization Inventory.

This prospective, double-blind, randomized clinical trial was designed as a parallel-group study with balanced randomization and enrolled 137 patients scheduled to undergo TKA surgery performed by one operator at a hospital from March 2019 to February 2020. All patients were screened using the Central Sensitization Inventory (CSI) 2 weeks before surgery, where a CSI score above 40 points indicated CS [28]. Patients who met all of the following standards were allowed to participate in this study: (1) surgery performed for primary knee OA; (2) American Society of Anesthesiologists (ASA) class of I, II, or III; (3) completion of the study informed consent form; and (4) more than 3 months of available follow-up data. Meanwhile, the exclusion criteria were as follows: (1) reasons for surgery other than primary OA, such as inflammatory arthritis (rheumatoid arthritis), osteonecrosis, or traumatic OA; (2) CSI score of fewer than 40 points; (3) prior use of duloxetine; (4) a known psychiatric disorder; and (5) previous infection history or previous operation history. Finally, among 137 TKA candidates, 39 CS patients were randomized and included in the final analysis (Figure 1). The study was approved by the appropriate institutional review board and posted at ClinicalTrials.gov.

The presence or absence of CS was assessed using CSI at the outpatient clinic 2 weeks before surgery in patients who were determined eligible to undergo TKA. CSI is a questionnaire designed for discriminating the established CS that has been validated; patients answer questions by themselves to discern whether they exhibit CS [29]. CSI consists of a total of 25 questions with scores ranging from zero to four points per question, with a total possible score of zero to 100 points [28]. According to the results of a previous study, patients with CS achieve CSI scores of 40 points or more [28].

Patients were randomly assigned to the duloxetine group or placebo group 2 weeks before surgery. For this study, patients were allocated in a block randomization method using a randomization program on the Internet. The patient allocation table was randomly extracted from a randomized envelope by an orthopedic surgeon who did not participate in the study, and the clinical pharmacy dispensed drugs without the knowledge of operators and evaluators. To confirm compliance with the clinical trial drug, the clinical pharmacy measured the amount of the drug returned by the patient. The patients in the duloxetine group took 30 mg of duloxetine once a day from 2 weeks before surgery to eight weeks after surgery, while the placebo group took the placebo medication once a day for the same period.

TKA was performed on all patients using the same posterior-stabilized implant by a single surgeon under general anesthesia with a tourniquet inflation level of 300 mmHg. A subvastus approach was adopted in all procedures. Before implantation, a multimodal intra-articular injection was performed and the implant was fixed using cement. For additional pain relief, a combination of ropivacaine, morphine, and ketorolac was used via periarticular injection, but steroids were not used due to the risk of infection [30]. A postoperative nerve block was not used either. After inserting the intra-articular suction drain in the joint, the capsule was repaired and wound closure was performed. Multimodal oral analgesic drugs containing 200 mg of celecoxib (Celebrex; Pfizer, New York, NY, USA) and 150 mg of pregabalin (Lyrica; Pfizer, New York, NY, USA) were administered 2 h before the surgery. All patients received intravenous patient-controlled analgesia (PCA) encompassing delivery of 1 mL of a 100 mL solution containing 2000 μg of fentanyl postoperatively. Each time the patient pressed the button, 1 mL of the drug was injected. Intravenous PCA was removed three days after surgery. Once patients restarted oral intake, 10 mg of oxycodone, 200 mg of celecoxib once daily, 37.5 mg of tramadol (Paramacet; Dong-A Pharm, Seoul, Korea), and 650 mg of acetaminophen (Tylenol; Janssen Korea, Seoul, Korea) were administered every 12 h for seven days during hospitalization. Intramuscular injections of tridol (Tramadol 50 mg, Yuhan Corp., Seoul, Korea) were administered as an acute analgesic therapy when a patient reported severe pain (>6 points) on the VAS (0–10 points). All patients performed active exercises according to the same rehabilitation protocol. Gradually increasing range-of-motion (ROM) and quadriceps-strengthening exercises were initiated immediately after the surgery. Patients began walking ambulation using a supportive device from the first postoperative day.

Active ROM of the knee joint was evaluated using a standard 60 cm goniometer with the patient in the supine position by one of the authors. To increase its accuracy, this measurement was made by one orthopedic specialist who did not otherwise participate in the study. PCA consumption during the 72 h following TKA was evaluated. Self-reported pain severity for resting, walking, nighttime, and 24 h average periods were measured using a 10-point VAS preoperatively; 1, 3, 5, and 7 days postoperative; and 2, 6, and 12 weeks postoperative. In the case of VAS, the doctor gave the patient a questionnaire and allowed the patient to indicate their pain level through an interview. The Brief Pain Inventory (BPI) was also deployed preoperatively and at 2, 6, and 12 weeks after surgery [31]. Among the BPI interference items, the sum of the relations with others, enjoyment of life, and mood and sleep subscores was defined as the affective subdimension of BPI, while the sum of walking, general activity, and work subscores was determined as the activity subdimension of BPI [13].

Postoperative wound complications were defined as occurring cases in which the patient needed additional treatment or procedures during the initial 12 weeks after surgery. Noted wound complications included wound dehiscence, suture granuloma, prolonged wound discharge continuing at 5 days after surgery, severe hematoma formation, or infection at the surgical site. Additional postoperative treatments or procedures included delayed discharge and readmission due to wound problems, additional outpatient visits to examine surgical wounds, topical application of ointments, surgical wound debridement or suturing at the hospital, hematoma aspiration, antibiotics prescription, and reoperation. Skin temperature was measured at the center of the patella using a portable digital thermometer (FLIR, Wilsonville, OR, USA) for the evaluation of surgical wound healing [32,33,34]. Skin temperature was evaluated preoperatively and 2, 6, and 12 weeks postoperatively between 9:00 and 12:00 a.m. Inflammatory markers including C-reactive protein (CRP) were evaluated preoperatively and 2 and 6 weeks after surgery. For cosmetic surgical wound evaluation, the surgical wound condition was compared using the Vancouver Scar Scale (VSS) at 12 weeks after surgery, which consists of four items, where pliability and height are scored from zero to four points, pigmentation and vascularity are scored from zero to three points, and the total score ranges between zero and 14 points. Importantly, when evaluating VSS results, the lower the score, the better the wound condition [35]. Wound score was evaluated by one orthopedic surgeon who did not participate in the surgery or research. Adverse events defined based on previous research into the safety of duloxetine were also evaluated during the follow-up period [26].

## 3. Statistical Analysis

No previous study has examined the effect of duloxetine on wound complications in CS patients undergoing TKA, so only the results of previous research examining wound complications in TKA patients with or without CS could be compared [21]. In patients who underwent TKA, wound complications occurred in 28.6% of the CS group and 2.7% of the non-CS group [21]. Therefore, the difference in the expected rate of wound complications between the duloxetine and placebo groups was set at 25% [21]. Next, the minimum sample size required for each group was calculated based on the findings of that study: having 34 patients in each group provided a statistical power of 80% and a level of 0.05. We chose a group size of 40 patients to allow for a dropout rate of up to 15%. All data are presented as mean and standard deviation values. The comparison of categorical variables between the two groups was assessed using Fisher’s exact test. An unpaired Student’s *t*-test was used for the analysis of continuous variables. ROM was assessed by repeated-measures analysis of variance (ANOVA). The Statistical Package for the Social Sciences version 21.0 (IBM Corporation, Armonk, NY, USA) was used for all statistical analyses. A *p*-value of less than 0.05 was considered to be statistically significant.

## 4. Results

**Figure 2:**
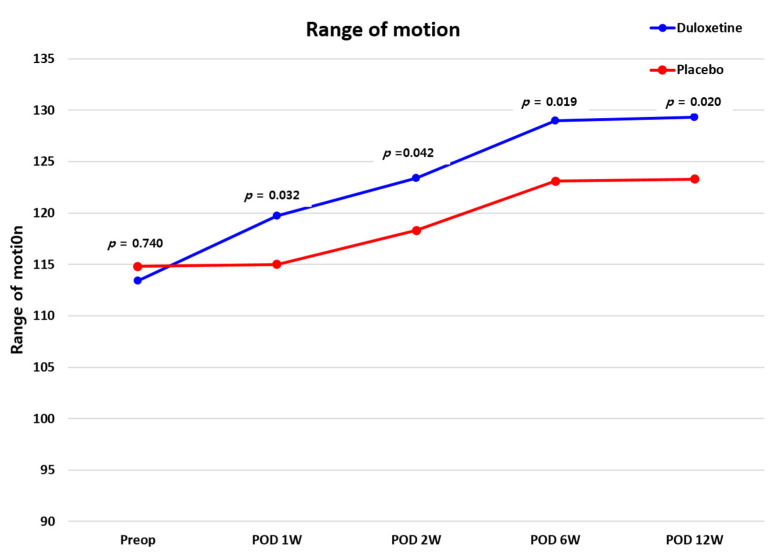
Comparison of ROM values between the duloxetine and placebo groups. ROM, range-of-motion.

To meet the originally intended sample size, we needed to recruit 80 patients (*n* = 40 per group). However, despite a one-year enrollment period, we were unable to recruit enough CS patients undergoing TKA. Because the contract with the department of clinical pharmacy at our hospital ended in April 2020, we needed to finish the study before that. Therefore, only 39 patients were enrolled in this study. There were no differences in demographic data recorded between the two groups before surgery (all *p* > 0.05) (Table 1). The CS score of the duloxetine group was 50.5 points, and that of the placebo group was 51.6 points (*p* > 0.05). Postoperatively, PCA consumption was 61.0 mL in the duloxetine group and 84.9 mL in the placebo group, a significant difference (*p =* 0.019). There was no difference in ROM between the two groups during the follow-up period (all *p* > 0.0125), (Figure 2).

**Figure 3:**
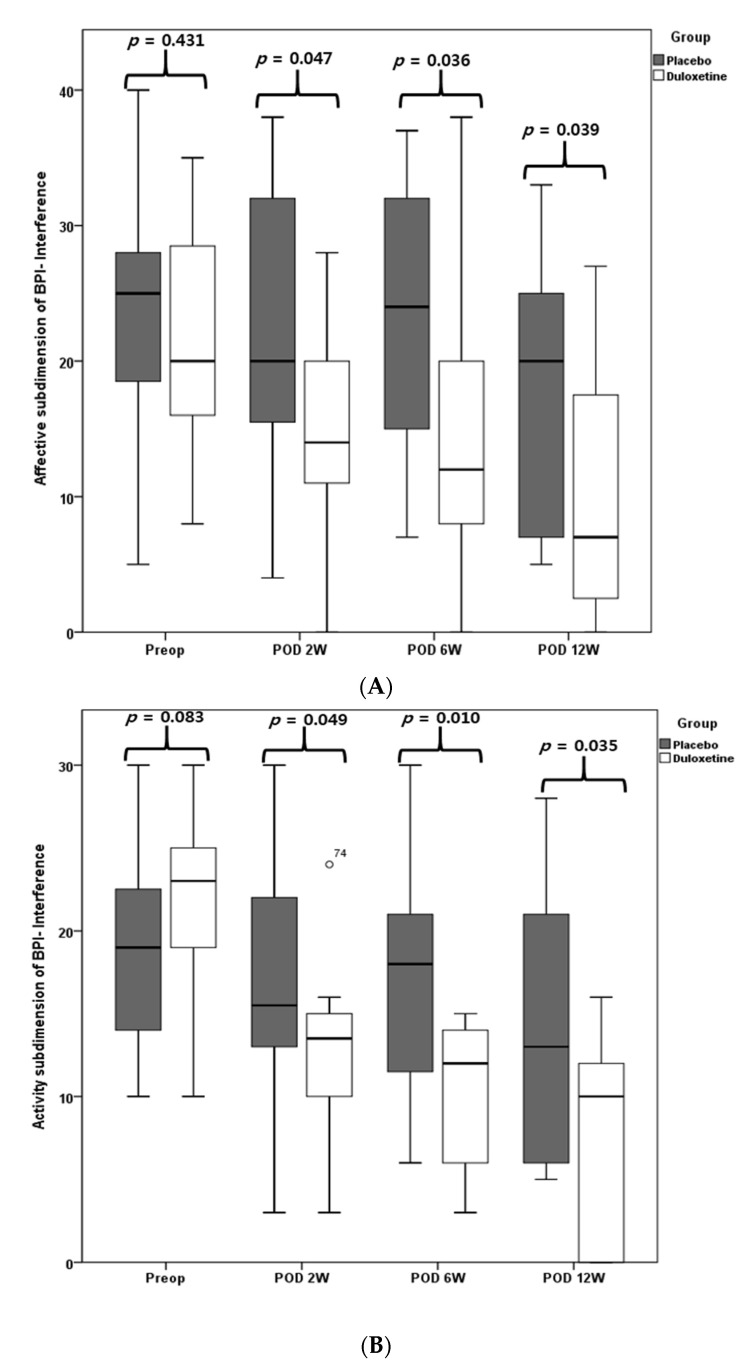
BPI interference. Affective subdimension of BPI-interference (**A**), activity subdimension of BPI interference (**B**).

There was no difference in VAS and BPI scores between the two groups before surgery (all *p* > 0.05); however, the duloxetine group presented significantly lower resting, walking, nighttime, and average VAS pain scores than the placebo group during follow-up periods of 1, 2, and 6 weeks after surgery (all *p* < 0.05) (Table 2). In terms of the affective and activity subdimensions of BPI interference, the duloxetine group showed significantly better results at 2, 6, and 12 weeks after surgery relative to the placebo group (all *p* < 0.05) (Figure 3).

Although the rate of wound complications was lower in the duloxetine group, this difference was not statistically significant (*p* > 0.05). Specifically, in the placebo group, aspiration was performed for hemarthrosis in one case, antibiotics were used in two cases due to wound redness, suture granuloma was found in two cases, and superficial surgical site infection occurred in one case, while, in the duloxetine group, antibiotics were used in one case due to wound redness. There was no need for reoperation or readmission during the follow-up period in both groups (Table 3).

**Figure 4:**
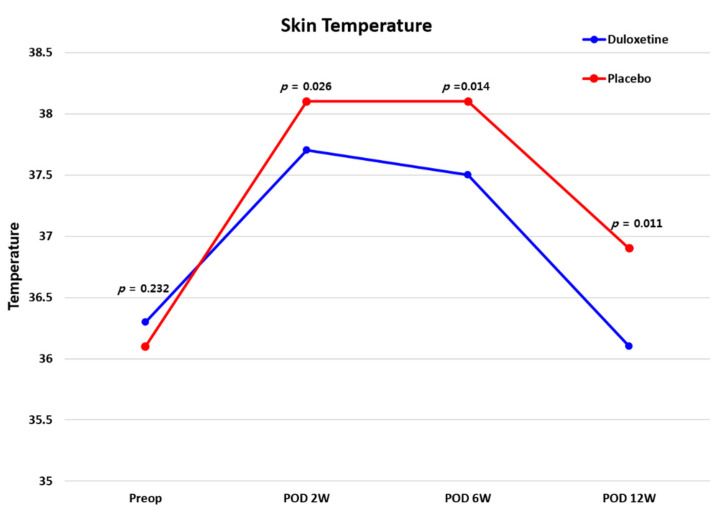
Telethermographic pattern of surgical wound healing after TKA between the two groups. TKA, total knee arthroplasty.

**Figure 5:**
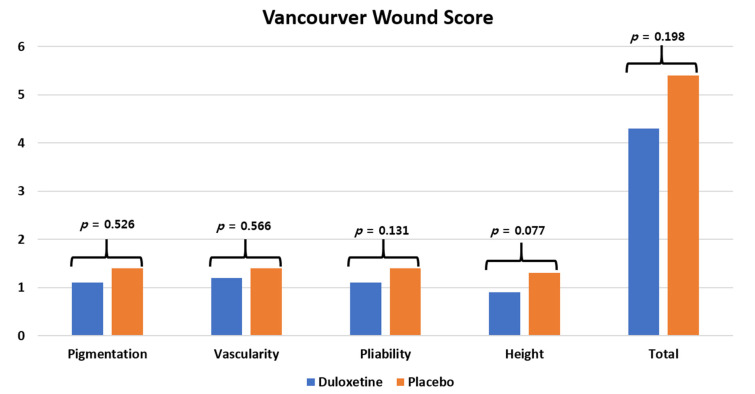
The Vancouver Scar Scale (VSS) score at 12 weeks postoperative.

As a result of measuring the changes in wound temperature using a thermal imaging camera, there was no difference found between the two groups before surgery (*p* > 0.05), but at 2, 6, and 12 weeks after surgery, the duloxetine group presented a significantly lower skin temperature than the control group (all *p* < 0.05) (Figure 4). There were no significant differences in CRP levels between the two groups during the follow-up period (all *p* > 0.05). Considering VSS scores, there was no significant difference between the two groups (all *p* > 0.05) (Figure 5), and the recorded adverse events also did not differ between the two groups (Table 4) (*p* > 0.05).

## 5. Discussion

The most important findings were that, among CS patients who underwent TKA, those who took duloxetine from 2 weeks before surgery to 8 weeks after experienced less pain and superior functionality until three months after surgery, and their wound temperature was also significantly lower as compared with patients in the placebo group.

The expiration date for drug storage and availability was April 2020, so no additional patient enrollment was performed. Still, a sample size of 39 patients is sufficient to evaluate the feasibility of the study design. According to the rule of thumb published previously for the feasibility study, a sample size of 12 or more participants per group is determined to be sufficient [36]. Koh et al. [13] reported that duloxetine offered effective pain control and functional recovery for CS patients undergoing TKA, and Kim et al. [21] reported that patients with CS had a higher incidence of wound complications than those without CS. To the best of our knowledge, no previous prospective study examined whether duloxetine could reduce postoperative pain and the incidence of wound complications in CS patients undergoing TKA. In this study, the 12-week period was too short to enroll enough CS patients, so our data are underpowered to detect differences in wound complications. However, our results still have meaning as a report. To clearly answer our research questions, we intend to conduct further research involving a larger sample enrolled across a longer period of more than 1 year.

Surgical stress response describes the physiological and pathophysiological changes induced by surgical stimulation. It can be broadly divided into neuroendocrine–metabolic and inflammatory–immune responses [6,37]. Postoperative sensitization of the central neurons may result in hyperalgesia and allodynia in surgical patients [38]. This is a typical symptom of CS, and surgical response stress may be greater in CS patients [7,8]. In order to modulate the surgical stress response, various drugs for anesthesia and analgesia are used in combination [6]. Duloxetine is a well-known effective agent for CS [13,26], and the use of duloxetine in CS patients mitigates surgical stress response through CS mitigation and localized factors that reduce postoperative pain and help wound healing [13,21,26,27].

Although OA pain has traditionally been regarded as peripheral and nociceptive pain caused by inflammation or mechanical damage to peripheral tissues, new evidence suggests that CS is also an important underlying mechanism of OA pain [39,40]. CS is accompanied by impaired activity of the descending inhibitory pathway [41]. Serotonin and norepinephrine are key neurotransmitters in the descending inhibitory pathway and are involved in pain control [24]. Duloxetine is a centrally acting analgesic that enhances the activity of descending inhibitory pain pathways [26,42], particularly dysfunctional inhibitory pain pathways, and specifically decreases descending pathway activity in patients with CS [43]. Since this study targeted patients with CS, duloxetine was selected as the study drug because it was considered most suitable for knee OA patients with CS. Generally, the dosage of duloxetine is initially started at 30 mg and then increased to 60 mg because duloxetine may have side effects [44]. In previous studies, 30 mg of duloxetine showed a sufficient effect [13,45]. Therefore, in this study, 30 mg of duloxetine was used continuously without dose adjustment.

In CS patients, duloxetine, which was used as part of preemptive analgesia and perioperative pain management, achieved a better pain control effect after TKA surgery. In addition, it also demonstrated sufficient advantages regarding opioid consumption after surgery. The reason for using duloxetine from 2 weeks before surgery for the purpose of preemptive analgesia is that, in the previous study, the effect of duloxetine on pain in CS patients who underwent TKA appeared from 2 weeks after surgery [13]. Therefore, it was judged that setting a period of 2 weeks was appropriate for evaluating the degree of pain control immediately after surgery. There have been various investigations performed into duloxetine as part of a perioperative pain-management regimen after TKA [46,47], but existing studies on the effects of duloxetine on pain reduction before and after surgery in CS patients are largely insufficient [13]. In the previous studies, perioperative use of duloxetine was effective in reducing opioid consumption, but not in reducing pain [46,47]. However, those results were from patients who underwent TKA without CS screening. Thus, they have limited value in assessing the effects of duloxetine for pain reduction in CS patients. Information was lacking because insufficient studies have examined the effect of duloxetine in CS patients undergoing TKA. A high-level study investigated the pain-reduction effect by having CS patients undergoing TKA take duloxetine from one day before surgery to 6 weeks after surgery and reported that duloxetine achieved a significant pain-reduction effect from 2 weeks after surgery [13]. Nevertheless, there are limitations to analyzing the effects of preemptive duloxetine on pain reduction immediately after surgery in terms of not having enough duloxetine administered before surgery. Since CS is known to be an important risk factor for persistent postoperative pain in TKA patients [12,13,14,15], the evidence from this study supports the use of duloxetine as a preoperative multimodal pain-management protocol when performing TKA surgery in CS patients.

Serotonin plays an important role in the promotion of wound healing, especially in the inflammatory and proliferative phases [1,9]. In wound healing, serotonin promotes platelet activation and hemostasis by stimulating the secretion of von Willebrand factor (*vWf*) from endothelial cells [9]. In addition, serotonin can mitigate chronic inflammation or hypertrophic scarring by promoting the secretion of pro-inflammatory cytokines and inhibiting the apoptosis of human monocytes [10]. Norepinephrine is also associated with the wound healing process. In a comparison of wound healing in norepinephrine-intact and -depleted mice, neutrophil infiltration was more common in norepinephrine-intact mice, and re-epithelialization was also accelerated. Norepinephrine plays an important role in wound healing and infection defense by regulating the inflammatory and proliferative phases of wound healing and improving the recruitment and wound closure effects of innate immune cells [2]. CS involves various mechanisms, one of which is a decrease in the descending inhibition pathway [16,17]. The decreased function of that descending pathway is associated with a reduction in the role of serotonin [21]. Malinin et al. suggested that a serotonin reuptake inhibitor can promote wound healing by sustaining an extracellular concentration of serotonin through its inhibition of the serotonin transporter [8]. In a study by Li et al., fluoxetine (a serotonin reuptake inhibitor) significantly reduced skin lesions and scratching [7]. Sufficient evidence supports the ability of serotonin–norepinephrine reuptake inhibitors to promote wound healing.

In addition, pain is also closely related to wound healing [48,49]. The difference in pain between the duloxetine group and the placebo group in CS patients is an important factor that can affect wound healing. The incidence rate of wound complication was 30% in the duloxetine group and 5% in the placebo group in CS patients who underwent TKA, respectively. In a study by Kim et al., the rate of wound complications in the CS group was 28.6%, which was much higher than that of the non-CS group (2.7%) [21]. In this study, the wound complications rate of the CS patient group taking the placebo was 30%, which was similar to the previous study results. The wound complication rate was lower in the duloxetine group, but that difference was not statistically significant. In addition, since the CS group had a 15-fold higher risk of wound complications than the non-CS group [21], it is essential to study the effect of duloxetine on wound complications in CS patients using a larger sample size.

Infrared thermal imaging (IRT) detects temperature changes associated with various diseases [50,51] and is useful for the postoperative monitoring of surgical site wound healing [52,53]. Even after TKA, IRT has been used for the monitoring of wound healing and has proven to be a useful imaging modality [32,33]. In fact, at the surgical site, changes in skin temperature occur due to the postoperative inflammatory response as part of the postoperative healing process [34]. In this study, the duloxetine group showed significantly lower skin temperatures than the placebo group at 2, 6, and 12 weeks after surgery. Notably, the skin temperature had almost reached the preoperative level by 12 weeks postoperative in the duloxetine group, while the placebo group showed higher skin temperatures than those recorded before surgery even at 12 weeks after surgery. Skin temperature is a sensitive measurement tool that reflects the local inflammatory response [54]; thus, based on these results, it can be confirmed that duloxetine helps promote wound healing at the surgical site in CS patients undergoing TKA.

Preoperative and postoperative use of duloxetine relative to placebo therapy did not increase adverse events in patients with CS who underwent TKA. This is consistent with the findings of previous studies of TKA patients using duloxetine to confirm their safety [46,47]. Since the study sample size was small, there may be limitations in showing clinical relevance, but the frequency of side effects in the group taking duloxetine was similar to the previous results [46,47]. However, additional studies are needed to confirm the safety while using duloxetine during preemptive and perioperative periods in CS patients undergoing TKA.

This study has several limitations that should be noted. First, most of the participants were women. It is well known that women make up the majority of the Korean TKA population [55,56,57]. Second, our sample size was small, so our research might be underpowered, including the possibility of type 2 errors in all relevant outcomes. A larger study might be necessary to develop a general recommendation for the use of preemptive duloxetine in TKA patients with CS. Third, the most accurate way to diagnose a psychiatric disorder is to use a screening tool such as the Hospital Anxiety and Depression Scale [1]. However, in this study, psychiatric disorders were determined using patient responses as they gave their medical history. In addition, patients with a psychiatric history may receive greater benefit from duloxetine, because psychiatric factors are closely related to CS [58]. The purpose of this study was to investigate the effect of duloxetine on CS-related knee OA. For this reason, we sought to exclude cases of diseases other than knee OA related to CS. Fourth, wound size was not evaluated in this study. Fifth, in CS, disease is not limited to just the knee joint, so research should be expanded to other parts of the body [59]. Finally, the follow-up period was relatively short at three months after surgery. The long-term influence of duloxetine on postoperative wound complications and postoperative pain patterns could not be evaluated in this study and should be explored in a study with a longer follow-up period. Despite these limitations, however, this study is of great significance in that it provides valuable information for the first time on the effects of preemptive and perioperative duloxetine on postoperative pain levels and wound healing in CS patients who underwent TKA.

## 6. Conclusions

In this study, preemptive duloxetine effectively reduced pain and lowered wound temperature immediately after surgery in CS patients subjected to TKA. Based on these results, further study involving larger populations is warranted to clarify the effects of duloxetine on postoperative pain and wound healing in CS patients undergoing TKA.

## Figures and Tables

**Table 1:** Demographic characteristics of the two groups.

	Placebo (*n* = 20)	Duloxetine (*n* = 19)	*p*-Value
Demographics			
Age (years)	67.0 (7.1)	71.2 (6.5)	0.055
Gender (Female, %)	16 (80.0%)	17 (89.5%)	0.412
BMI (kg/m^2^)	25.7 (3.4)	25.7 (4.5)	0.978
Operation side (Left, %)	6 (30.0%)	5 (26.3%)	0.798
CSI score	51.6 (11.6)	50.5 (11.5)	0.772
ASA grade			0.333
1	7 (35.0%)	4 (21.1%)	
2	13 (65.0%)	15 (78.9%)	
Tourniquet time (minutes)	42.0 (8.1)	43.3 (8.4)	0.678
Specific comorbidities			
Hypertension	9 (45.0%)	11 (57.9%)	0.421
Diabetes	2 (10.0%)	4 (21.1%)	0.339
Cardiac disease	1 (5.0%)	4 (21.1%)	0.134
Cerebrovascular event	2 (10.0%)	2 (10.5%)	0.957
Thyroid disease	0 (0%)	0 (0%)	1.000
Kidney disease	1 (5.0%)	1 (5.3%)	0.970
Pulmonary disease	1 (5.0%)	1 (5.3%)	0.970
Liver disease	1 (5.0%)	0 (0%)	0.323
Hemovac drainage (mL)	208.2 (145.7)	189.0 (123.1)	0.661

BMI, Body Mass Index; ASA, American Society of Anesthesiologists; CSI, Central Sensitization Inventory. The values are presented as mean and SD with the range in parentheses.

**Table 2:** Pain Visual Analogue Scale on resting, walking, nighttime, and 24 h average *.

	Resting			Walking			Nighttime			24 h Average		
	Control (*n* = 20)	Duloxetine (*n* = 19)	*p*-Value	Control (*n* = 20)	Duloxetine (*n* = 19)	*p*-Value	Control (*n* = 20)	Duloxetine (*n* = 19)	*p*-Value	Control (*n* = 20)	Duloxetine (*n* = 19)	*p*-Value
Preop	3.8 (1.9)	4.4 (1.8)		4.9 (1.8)	5.8 (1.4)		5.7 (1.1)	5.6 (1.2)		6.1 (1.0)	5.6 (1.8)	
PO 1D	5.7 (1.4)	3.5 (1.4)	<0.001	7.3 (1.4)	5.0 (1.6)	<0.001	4.9 (1.7)	6.2 (2.0)	<0.001	5.5 (1.5)	4.7 (1.3)	<0.001
PO 3D	5.2 (1.5)	3.8 (2.0)	0.029	6.6 (1.0)	5.0 (2.2)	0.009	6.4 (2.0)	5.1 (2.1)	0.091	5.4 (1.3)	4.2 (1.4)	0.015
PO 5D	4.6 (1.3)	2.8 (1.5)	0.001	6.1 (1.8)	4.5 (1.3)	0.009	6.1 (2.0)	3.9 (1.8)	0.002	5.5 (1.1)	3.4 (0.8)	0.001
PO 1W	4.3 (1.6)	2.5 (1.3)	0.001	6.0 (1.6)	4.1 (1.2)	0.001	5.8 (2.3)	3.5 (1.9)	0.004	5.2 (1.3)	3.8 (1.7)	0.012
PO 2W	4.3 (1.5)	2.8 (1.2)	0.002	5.4 (1.4)	4.1 (1.6)	0.016	5.8 (1.4)	4.2 (1.5)	0.002	5.1 (1.5)	3.7 (1.5)	0.008
PO 6W	3.3 (1.7)	1.9 (1.2)	0.014	4.0 (1.7)	2.4 (1.6)	0.011	4.6 (1.7)	2.8 (1.6)	0.002	3.7 (1.6)	2.3 (1.2)	0.006
PO 12W	2.8 (1.0)	2.2 (1.3)	0.205	3.4 (0.8)	2.6 (1.3)	0.068	3.7 (0.8)	2.9 (1.3)	0.057	3.1 (1.1)	2.5 (1.2)	0.187

* Data are presented as mean (standard deviation). PO = postoperative; D = day; W = week.

**Table 3:** Intergroup comparison of wound complications.

	Placebo (*n* = 20)	Duloxetine (*n* = 19)	*p*-Value
Wound complication	6 (30.0%)	1 (5.3%)	0.091
Hematoma aspiration	1 (5.0%)	0 (0%)	
Drainage occurring after postoperative day 5	0 (0%)	0 (0%)	
Suture granuloma	2 (10.0%)	0 (0%)	
Additional antibiotics for redness	2 (10.0%)	1 (5.3%)	
Superficial surgical site infection	1 (5.0%)	0 (2.0%)	

**Table 4:** Incidence rates of adverse events *.

Adverse Events	Placebo (*n* = 20)	Duloxetine (*n* = 19)	*p*-Value
Nausea/vomiting	6 (30.0)	3 (15.8)	0.292
Dizziness	5 (25.0)	6 (31.6)	0.648
Insomnia	7 (35.0)	10 (52.6)	0.267
Fatigue	4 (20.0)	7 (36.8)	0.243
Appetite loss	8 (40.0)	9 (47.4)	0.643
Dry mouth	10 (50.0)	5 (26.3)	0.129
Constipation	12 (60.0)	10 (52.6)	0.643

* Data are presented as number (percentage) of patients.

## Data Availability

Data collected for this study, including individual patient data, will not be made.

## References

[1] Losina E., Thornhill T.S., Rome B.N., Wright J., Katz J.N. (2012). The dramatic increase in total knee replacement utilization rates in the United States cannot be fully explained by growth in population size and the obesity epidemic. J. Bone Jt. Surg. Am. Vol..

[2] Beswick A.D., Wylde V., Gooberman-Hill R., Blom A., Dieppe P. (2012). What proportion of patients report long-term pain after total hip or knee replacement for osteoarthritis? A systematic review of prospective studies in unselected patients. BMJ Open.

[3] Scott C.E., Howie C.R., MacDonald D., Biant L.C. (2010). Predicting dissatisfaction following total knee replacement: A prospective study of 1217 patients. J. Bone Jt. Surg. Br. Vol..

[4] Kehlet H. (1997). Multimodal approach to control postoperative pathophysiology and rehabilitation. Br. J. Anaesth..

[5] Andersen L., Husted H., Kristensen B.B., Otte K.S., Gaarn-Larsen L., Kehlet H. (2010). Analgesic efficacy of subcutaneous local anaesthetic wound infiltration in bilateral knee arthroplasty: A randomised, placebo-controlled, double-blind trial. Acta Anaesthesiol. Scand..

[6] Cusack B., Buggy D.J. (2020). Anaesthesia, analgesia, and the surgical stress response. BJA Educ..

[7] Woolf C.J. (2011). Central sensitization: Implications for the diagnosis and treatment of pain. Pain.

[8] Woolf C.J., Chong M.S. (1993). Preemptive analgesia—Treating postoperative pain by preventing the establishment of central sensitization. Anesth. Analg..

[9] Hochman J.R., Gagliese L., Davis A.M., Hawker G.A. (2011). Neuropathic pain symptoms in a community knee OA cohort. Osteoarthr. Cartil..

[10] Ohtori S., Orita S., Yamashita M., Ishikawa T., Ito T., Shigemura T., Nishiyama H., Konno S., Ohta H., Takaso M. (2012). Existence of a neuropathic pain component in patients with osteoarthritis of the knee. Yonsei Med. J..

[11] Valdes A.M., Suokas A.K., Doherty S.A., Jenkins W., Doherty M. (2014). History of knee surgery is associated with higher prevalence of neuropathic pain-like symptoms in patients with severe osteoarthritis of the knee. Semin. Arthritis Rheum..

[12] Kim S.H., Yoon K.B., Yoon D.M., Yoo J.H., Ahn K.R. (2015). Influence of Centrally Mediated Symptoms on Postoperative Pain in Osteoarthritis Patients Undergoing Total Knee Arthroplasty: A Prospective Observational Evaluation. Pain Pract. Off. J. World Inst. Pain.

[13] Koh I.J., Kim M.S., Sohn S., Song K.Y., Choi N.Y., In Y. (2019). Duloxetine Reduces Pain and Improves Quality of Recovery Following Total Knee Arthroplasty in Centrally Sensitized Patients: A Prospective, Randomized Controlled Study. J. Bone Jt. Surg. Am. Vol..

[14] Lundblad H., Kreicbergs A., Jansson K.A. (2008). Prediction of persistent pain after total knee replacement for osteoarthritis. J. Bone Jt. Surg. Br. Vol..

[15] Wylde V., Palmer S., Learmonth I.D., Dieppe P. (2013). The association between pre-operative pain sensitisation and chronic pain after knee replacement: An exploratory study. Osteoarthr. Cartil..

[16] Latremoliere A., Woolf C.J. (2009). Central sensitization: A generator of pain hypersensitivity by central neural plasticity. J. Pain Off. J. Am. Pain Soc..

[17] Lee Y.C., Nassikas N.J., Clauw D.J. (2011). The role of the central nervous system in the generation and maintenance of chronic pain in rheumatoid arthritis, osteoarthritis and fibromyalgia. Arthritis Res. Ther..

[18] Duerschmied D., Suidan G.L., Demers M., Herr N., Carbo C., Brill A., Cifuni S.M., Mauler M., Cicko S., Bader M. (2013). Platelet serotonin promotes the recruitment of neutrophils to sites of acute inflammation in mice. Blood.

[19] Gosain A., Jones S.B., Shankar R., Gamelli R.L., DiPietro L.A. (2006). Norepinephrine modulates the inflammatory and proliferative phases of wound healing. J. Trauma.

[20] Schlüter T., Bohnensack R. (1999). Serotonin-induced secretion of von Willebrand factor from human umbilical vein endothelial cells via the cyclic AMP-signaling systems independent of increased cytoplasmic calcium concentration. Biochem. Pharm..

[21] Kim M.S., Koh I.J., Lee S.Y., In Y. (2018). Central sensitization is a risk factor for wound complications after primary total knee arthroplasty. Knee Surg. Sports Traumatol. Arthrosc. Off. J. Esska.

[22] Garbedian S., Sternheim A., Backstein D. (2011). Wound healing problems in total knee arthroplasty. Orthopedics.

[23] Vince K., Chivas D., Droll K.P. (2007). Wound complications after total knee arthroplasty. J. Arthroplast..

[24] Fields H.L., Heinricher M.M., Mason P. (1991). Neurotransmitters in nociceptive modulatory circuits. Annu. Rev. Neurosci..

[25] Woolf C.J. (2004). Pain: Moving from symptom control toward mechanism-specific pharmacologic management. Ann. Intern. Med..

[26] Chappell A.S., Ossanna M.J., Liu-Seifert H., Iyengar S., Skljarevski V., Li L.C., Bennett R.M., Collins H. (2009). Duloxetine, a centrally acting analgesic, in the treatment of patients with osteoarthritis knee pain: A 13-week, randomized, placebo-controlled trial. Pain.

[27] Malinin A., Oshrine B., Serebruany V. (2004). Treatment with selective serotonin reuptake inhibitors for enhancing wound healing. Med. Hypotheses.

[28] Neblett R., Cohen H., Choi Y., Hartzell M.M., Williams M., Mayer T.G., Gatchel R.J. (2013). The Central Sensitization Inventory (CSI): Establishing clinically significant values for identifying central sensitivity syndromes in an outpatient chronic pain sample. J. Pain Off. J. Am. Pain Soc..

[29] Mayer T.G., Neblett R., Cohen H., Howard K.J., Choi Y.H., Williams M.J., Perez Y., Gatchel R.J. (2012). The development and psychometric validation of the central sensitization inventory. Pain Pract. Off. J. World Inst. Pain.

[30] Kim T.W., Park S.J., Lim S.H., Seong S.C., Lee S., Lee M.C. (2015). Which analgesic mixture is appropriate for periarticular injection after total knee arthroplasty? Prospective, randomized, double-blind study. Knee Surg. Sports Traumatol. Arthrosc. Off. J. Esska.

[31] Cleeland C.S., Ryan K.M. (1994). Pain assessment: Global use of the Brief Pain Inventory. Ann. Acad. Med. Singap..

[32] Honsawek S., Deepaisarnsakul B., Tanavalee A., Sakdinakiattikoon M., Ngarmukos S., Preativatanyou K., Bumrungpanichthaworn P. (2011). Relationship of serum IL-6, C-reactive protein, erythrocyte sedimentation rate, and knee skin temperature after total knee arthroplasty: A prospective study. Int. Orthop..

[33] Romanò C.L., Romanò D., Dell’Oro F., Logoluso N., Drago L. (2011). Healing of surgical site after total hip and knee replacements show similar telethermographic patterns. J. Orthop. Traumatol. Off. J. Ital. Soc. Orthop. Traumatol..

[34] Xu X., Sang W., Liu Y., Zhu L., Lu H., Ma J. (2018). Effect of Celecoxib on Surgical Site Inflammation after Total Knee Arthroplasty: A Randomized Controlled Study. Med. Princ. Pract. Int. J. Kuwait Univ. Health Sci. Cent..

[35] Baryza M.J., Baryza G.A. (1995). The Vancouver Scar Scale: An administration tool and its interrater reliability. J. Burn Care Rehabil..

[36] Julious S.A. (2005). Sample size of 12 per group rule of thumb for a pilot study. Pharm. Stat. J. Appl. Stat. Pharm. Ind..

[37] Manou-Stathopoulou V., Korbonits M., Ackland G.L. (2019). Redefining the perioperative stress response: A narrative review. Br. J. Anaesth..

[38] Dahl J.B., Erichsen C.J., Fuglsang-Frederiksen A., Kehlet H. (1992). Pain sensation and nociceptive reflex excitability in surgical patients and human volunteers. Br. J. Anaesth..

[39] Arendt-Nielsen L., Nie H., Laursen M.B., Laursen B.S., Madeleine P., Simonsen O.H., Graven-Nielsen T. (2010). Sensitization in patients with painful knee osteoarthritis. Pain.

[40] Fingleton C., Smart K., Moloney N., Fullen B.M., Doody C. (2015). Pain sensitization in people with knee osteoarthritis: A systematic review and meta-analysis. Osteoarthr. Cartil..

[41] Tracey I., Mantyh P.W. (2007). The cerebral signature for pain perception and its modulation. Neuron.

[42] Chappell A.S., Desaiah D., Liu-Seifert H., Zhang S., Skljarevski V., Belenkov Y., Brown J.P. (2011). A double-blind, randomized, placebo-controlled study of the efficacy and safety of duloxetine for the treatment of chronic pain due to osteoarthritis of the knee. Pain Pract. Off. J. World Inst. Pain.

[43] Smith H.S., Smith E.J., Smith B.R. (2012). Duloxetine in the management of chronic musculoskeletal pain. Clin. Risk Manag..

[44] Blikman T., Rienstra W., van Raaij T.M., ten Hagen A.J., Dijkstra B., Zijlstra W.P., Bulstra S.K., van den Akker-Scheek I., Stevens M. (2016). Duloxetine in OsteoArthritis (DOA) study: Study protocol of a pragmatic open-label randomised controlled trial assessing the effect of preoperative pain treatment on postoperative outcome after total hip or knee arthroplasty. BMJ Open.

[45] Boudreau D., Von Korff M., Rutter C.M., Saunders K., Ray G.T., Sullivan M.D., Campbell C.I., Merrill J.O., Silverberg M.J., Banta-Green C. (2009). Trends in long-term opioid therapy for chronic non-cancer pain. Pharm. Drug Saf..

[46] Ho K.Y., Tay W., Yeo M.C., Liu H., Yeo S.J., Chia S.L., Lo N.N. (2010). Duloxetine reduces morphine requirements after knee replacement surgery. Br. J. Anaesth..

[47] YaDeau J.T., Brummett C.M., Mayman D.J., Lin Y., Goytizolo E.A., Padgett D.E., Alexiades M.M., Kahn R.L., Jules-Elysee K.M., Fields K.G. (2016). Duloxetine and Subacute Pain after Knee Arthroplasty when Added to a Multimodal Analgesic Regimen: A Randomized, Placebo-controlled, Triple-blinded Trial. Anesthesiology.

[48] Beilin B., Shavit Y., Trabekin E., Mordashev B., Mayburd E., Zeidel A., Bessler H. (2003). The effects of postoperative pain management on immune response to surgery. Anesth. Analg..

[49] McGuire L., Heffner K., Glaser R., Needleman B., Malarkey W., Dickinson S., Lemeshow S., Cook C., Muscarella P., Melvin W.S. (2006). Pain and wound healing in surgical patients. Ann. Behav. Med. A Publ. Soc. Behav. Med..

[50] Nogueira F.E., Medeiros F., Barroso L.V., Miranda E.P., de Castro J.D., Mota Filho F.H. (2009). Infrared digital telethermography: A new method for early detection of varicocele. Fertil. Steril..

[51] Ring E.F. (2006). The historical development of thermometry and thermal imaging in medicine. J. Med. Eng. Technol..

[52] Horzic M., Bunoza D., Maric K. (1996). Contact Thermography in a study of primary healing of surgical wounds. Ostomy Wound Manag..

[53] Horzic M., Bunoza D., Maric K. (1996). Three-dimensional observation of wound temperature in primary healing. Ostomy Wound Manag..

[54] Romanò C.L., Logoluso N., Dell’Oro F., Elia A., Drago L. (2012). Telethermographic findings after uncomplicated and septic total knee replacement. Knee.

[55] Kim C.W., Lee C.R. (2018). Effects of Femoral Lateral Bowing on Coronal Alignment and Component Position after Total Knee Arthroplasty: A Comparison of Conventional and Navigation-Assisted Surgery. Knee Surg. Relat. Res..

[56] Kim S.H., Park Y.B., Song M.K., Lim J.W., Lee H.J. (2018). Reliability and Validity of the Femorotibial Mechanical Axis Angle in Primary Total Knee Arthroplasty: Navigation versus Weight Bearing or Supine Whole Leg Radiographs. Knee Surg. Relat. Res..

[57] Yoo J.H., Oh H.C., Park S.H., Kim J.K., Kim S.H. (2018). Does Obesity Affect Clinical and Radiological Outcomes in Minimally Invasive Total Knee Arthroplasty? Minimum 5-Year Follow-up of Minimally Invasive TKA in Obese Patients. Clin. Orthop. Surg..

[58] Adams L.M., Turk D.C. (2015). Psychosocial factors and central sensitivity syndromes. Curr. Rheumatol. Rev..

[59] Staud R. (2011). Evidence for shared pain mechanisms in osteoarthritis, low back pain, and fibromyalgia. Curr. Rheumatol. Rep..

